# Influence of Must Clarification Technique on the Volatile Composition of Albariño and Treixadura Wines

**DOI:** 10.3390/molecules27030810

**Published:** 2022-01-26

**Authors:** Iván Vázquez-Pateiro, José Manuel Mirás-Avalos, Elena Falqué

**Affiliations:** 1 Departamento de Química Analítica, Facultad de Ciencias, Universidade de Vigo, As Lagoas s/n, 32004 Ourense, Spain; ivpateiro@yahoo.es; 2 Departamento de Sistemas Agrícolas, Forestales y Medio Ambiente, Centro de Investigación y Tecnología Agroalimentaria de Aragón (CITA), Avda. Montañana 930, 50059 Zaragoza, Spain

**Keywords:** white wine, must clarification, static settling, flotation, volatile compounds

## Abstract

Clarification of the musts is carried out to remove particles that cause turbidity, oxidizable polyphenols, and eliminate excess of proteins. However, an excessive clarification of the musts can lead to the reduction of volatile compound concentrations and, as a consequence, modify the sensorial properties of the wines. Therefore, in this study, the influence of two pre-fermentation clarification techniques (static settling and flotation) on the concentrations of volatile compounds has been assessed in Albariño and Treixadura wines. Fermentations were performed at an industrial scale. Volatile compounds have been identified and quantified by gas chromatography (FID and mass spectrometry detection) and expert panelists assessed the sensory properties of the final wines. The results showed effects of the clarification techniques on the volatile composition of wines from both varieties. Flotation significantly increased the concentrations of benzyl alcohol in Treixadura wines, whereas this technique increased the concentration of 1-hexanol, octanoic acid, and furfural in Albariño wines, but without exceeding the corresponding perception thresholds. Panelists tended to score higher the wines coming from flotation, which, together with the shorter application time, makes this technique suitable for clarifying the musts of these two white varieties.

## 1. Introduction

The interaction of hundreds of chemical compounds produces the aroma of wines [1]. According to their origin, these compounds derive directly from grapes such as norisoprenoids and monoterpenes [2,3]; secondary metabolites released by the yeasts during fermentation (e.g., volatiles associated with the sugar and amino acid metabolism) [1,4]; and compounds related to wine storage in oak barrels or bottles [5,6]. The major groups of aroma compounds are monoterpenes, norisoprenoids, aliphatics, higher alcohols, esters, phenylpropanoids, methoxypyrazines, and volatile sulfur [2,7]. However, the character of the wine from a given grapevine variety does not relate to a single compound [1]. Therefore, the varietal character depends on the overall profile of odor-active compounds present in the grape and corresponding wine [1]. This character is extremely important for wine typicity and commercial success, as most wineries rely on this concept for marketing campaigns [8].

Grape solid particles contain some compounds, such as unsaturated and saturated fatty acids, phytosterols, nitrogen compounds, polysaccharides, etc., which are an important source of nutrients for yeasts during alcoholic fermentation [9]. The nature, size, and composition of these solids depend on the grape variety and the juice extraction process [10]. In white winemaking, the juices are usually clarified before fermentation. Among other factors, clarification of musts affects wine composition because this process removes suspended and colloidal particles that can cause instability or even health problems [11,12], but an excessive must clarification can exert negative effects, such as sluggish or unfinished fermentations and lower wine quality [13]. Therefore, it is necessary to reach a compromise between the minimum nutritional requirements for yeasts and the absence of unpleasant colors, odors, and flavors for white wine production [14]. Must stabilization and limpidity can be performed by sedimentation (natural or forced by centrifugation), filtration (macro-, micro-, or ultrafiltration), or flotation [11]. These processes are usually accompanied by a previous enzymatic pre-treatment [15], predominantly with pectinases. Another common enological operation performed to clarify musts or wines is the use of fining agents, which, alone or in combination with the techniques cited above, increase the settling efficiency, make the precipitation of suspended solids easier, minimize the browning, ensure stability, and improve the organoleptic characteristics, such as the modulation of mouthfeel perception or the reduction of off-flavors. These clarifying agents can have different origins: organic, inorganic, animal, or plant [11,16,17,18].

Albariño and Treixadura are white grapevine cultivars typical of Galicia (Northwest Spain) and the North of Portugal [19,20]. The wines produced with these cultivars have a high aromatic potential [21,22]. The volatile composition and sensory characteristics of wines made from these two cultivars have been previously reported [19,20,21,22,23,24,25]. The use of fining agents, in particular bentonite and silica gel in musts [26] and bentonite at different stages during vinification [27], has been previously studied in the Albariño variety. However, the effects of clarification techniques on volatile composition have never been determined in wines from these cultivars. Spontaneous (or natural) settling at low temperature is the most widely used pre-fermentative clarification process to remove insoluble materials from the grape must, mainly in white cultivars [28,29]. The must is left to settle for a few hours and the solid parts fall to the bottom of the tank, which is favored by lower temperatures and pectolytic enzymes [30]. However, this method is time consuming and some particles do not settle. Flotation can help to clarify grape musts more rapidly, because the solid parts adhere to the gas bubbles aided by somewhat higher temperatures, and the addition of clarifying agents that modify the must density, making the solid parts float and, therefore easier to remove [30]. In this context, the aim of the current study was to assess the potential modification of wine volatile composition and sensory profiles of monovarietal Albariño and Treixadura wines produced at an industrial scale when musts were submitted to clarification by flotation as compared to traditional static sedimentation.

## 2. Results

### 2.1. General Parameters of Wines as Affected by the Must Clarification Technique

Table 1 shows the general enological parameters for each wine obtained after the two clarification techniques applied to the musts for both Albariño and Treixadura varieties. Alcoholic content and volatile acidity were not significantly different (*p* < 0.05); however, pH values were significantly higher in wines obtained from musts subjected to flotation, and the total acidity showed mainly significantly lower values when the flotation was applied (Table 1). If the average behavior of each variety is considered, general parameters were not affected by the clarification technique before fermentation (Table 1). However, Treixadura wines tended to have lower alcoholic contents and total acidities when musts were clarified by flotation; however, these trends were not significant (*p*-values = 0.142 and 0.213, respectively, for alcohol content and total acidity).

### 2.2. Wine Volatile Composition as Affected by the Must Clarification Technique

A total of 56 volatiles were detected in the Albariño wine samples studied, including terpenes, norisoprenoids, C6 compounds, higher and other alcohols, acetates, ethyl esters, volatile fatty acids, volatile phenols, carbonyl compounds, and sulfur compounds (Table 2). Terpenes appeared at low concentrations and the most relevant volatile within this family was linalool. Among C6 compounds, 1-hexanol was the most quantitatively important volatile in all Albariño wines. Isoamyl alcohol and methanol were the most relevant alcohols detected in the Albariño wines studied. The most relevant ester was monoethyl succinate, while octanoic acid was the most quantitatively important fatty acid in the Albariño wines studied. Finally, the most relevant volatiles among acetates, carbonyl compounds, volatile phenols, and sulfur compounds were, respectively, isoamyl acetate, acetoine, 4-vinyl-phenol and methionol (Table 2).

In the case of Treixadura wines, a total of 53 volatiles were detected in the samples analyzed (Table 3). Terpenes were detected on a single sample of Treixadura wines and were present at very low concentrations, and β-damascenone and β-ionone were not detected. Similar to Albariño wines, 1-hexanol was the C6 compound detected at greater concentrations in Treixadura wines. In addition, isoamyl alcohol, and furfuryl alcohol, and methanol in some wines obtained from static sedimentation, were, quantitatively, the most important alcohols detected in Treixadura wines. Monoethyl succinate and octanoic acid were, respectively, the ester and fatty acid compounds that appeared at greater concentrations in Treixadura wines, but below that of Albariño wines. Finally, the acetate, carbonyl compound, volatile phenol, and sulfur compound most relevant were, respectively, isoamyl and benzyl acetates, acetoine, ethyl vanillate, and methionol (Table 3).

If the average behavior of each variety is considered, the must clarification technique showed a significant effect on three volatile compounds in Albariño wines: 1-hexanol, octanoic acid, and furfural (Table 4). Flotation significantly increased the concentrations of these volatiles in Albariño wines. In addition, a trend to greater concentrations (*p*-value = 0.087) of hexanoic acid in wines coming from musts that underwent a clarification treatment by flotation was observed. The must clarification treatment exerted a significant effect on the concentration of benzyl alcohol in Treixadura wines (Table 4), but this was not detected in Albariño wines. Flotation significantly increased the concentration of this volatile in Treixadura wines. In addition, a trend to higher concentrations of monoethyl succinate (*p*-value = 0.116) and benzaldehyde (*p*-value = 0.125) was observed in wines from the flotation treatment (Table 4).

**Figure 1:**
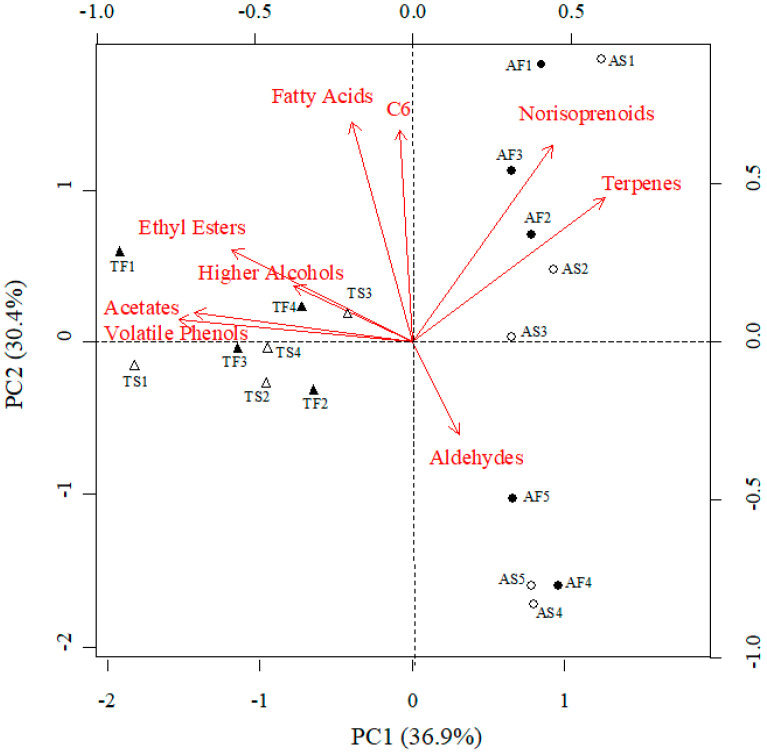
Bi-plot of the first two principal components (PC) for families of volatile compounds related to wine aroma of Albariño (circles) and Treixadura (triangles) wines coming from musts that underwent different clarification treatments: static clarification (open symbols) or flotation (full symbols). The label of each wine is provided close to its symbol (A stands for Albariño, T stands for Treixadura, S stands for Static settling, and F stands for Flotation).

Principal Component Analysis (PCA) applied to the cumulative concentrations of the different families of volatile compounds (Figure 1) explained 67.3% of the variability within the wine samples. The first component (PC1) explained 36.9% of this variability and depended mainly on the concentrations of volatile phenols, acetates, higher alcohols, and ethyl esters, while the PC2 accounted for 30.4% of the variability within the dataset and depended mainly on the concentrations of fatty acids, C6 compounds, norisoprenoids and terpenes (Figure 1). The PC1 was able to discriminate between Albariño and Treixadura samples, but no clear discrimination between the must clarification techniques could be achieved.

### 2.3. Wine Sensorial Properties as Affected by the Must Clarification Treatment

**Figure 2:**
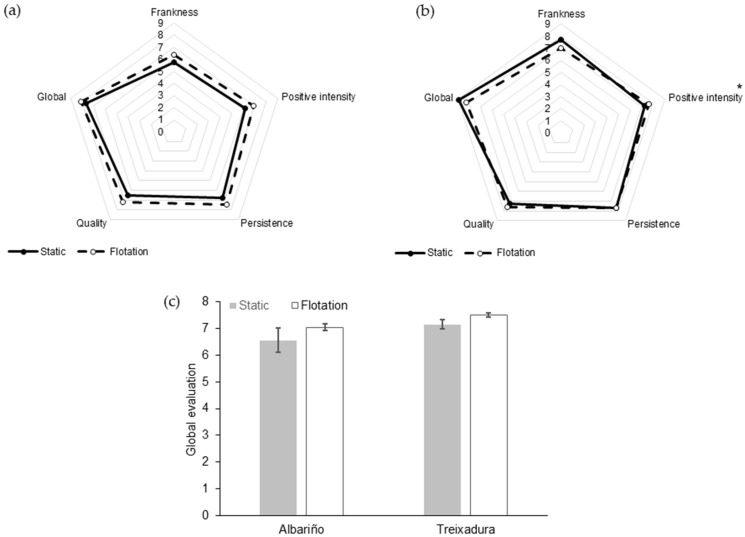
Sensory profile of Albariño (**a**) and Treixadura (**b**) wines as a function of the clarification treatment of the musts. Panel (**c**) displays the global mark given to the wines. The asterisk indicate significant differences (*p*-Value < 0.05) between treatments for a given descriptor.

The clarification treatment did not have a significant effect on the sensory perception of either Albariño or Treixadura wines (Figure 2). The only descriptor that showed a significant difference between treatments was the positive intensity in the mouth of the Treixadura wines (Figure 2b). The panelists tended to give higher marks of overall quality to the wines from the flotation treatment (Figure 2c), despite this difference was not statistically significant.

## 3. Discussion

Pre-fermentative clarification removes insoluble materials from the grape must, and added with fining agents or alone, can allow obtainment of fruity wines, reduce browning, and remove compounds that produce unwanted flavors [31]. However, it can have negative impacts on the composition of resulting wines depending on the technique employed [11,28,29].

The current study confirmed that clarification techniques based on physical processes, such as static settling and flotation, do not significantly affect the average general parameters of these white wines (Table 1), independently of the grapevine variety. This confirms previous research comparing static sedimentation vs. vacuum filtration [28,32], vs. turbidity adjusted by addition of part of the sediment [31], or vs. flotation [33]. Total and volatile acidities were lower in both wine varieties obtained from flotation, but not significantly different, and this behavior was also detected by Ma et al. [34] comparing the centrifugation and the membrane filtration in musts of Italian Riesling icewines vs. the wine control without any treatment. Conversely, Karagiannis and Lanaridis [35], in wines from three Greek varieties, and Albertin et al. [36], in Chardonnay wines, quantified higher volatile acidities when the musts showed lower turbidity, whereas the individual wines analyzed in this work showed lower volatile acidity, although only significantly different between clarification techniques in samples A1, A4, and T1 (Table 1). Except for T3 and T4 wines, all samples showed significantly different values according to the clarification technique, and wines with higher pH values showed simultaneous lower total acidity in both varieties (Table 1), in agreement with Karagiannis and Lanaridis [35]. Debina wines made from musts clarified by flotation using nitrogen (with and without pectolytic enzyme treatment) had lower total acidity than wines from must clarified by sedimentation [33].

Several studies reported alterations on the concentrations of wine volatile compounds depending on the clarification technique, either physical [28,37] or by the application of fining agents [26,34,38]. This could result in a loss of typicity, and alternatives have been proposed, such as the use of plant proteins [17] or the aforementioned physical methods. According to the data showed in Table 2 and Table 3, the concentrations of most of the volatile compounds significantly varied due to the clarification technique tested in this work. Moio et al. [38] showed that different clarification techniques (spontaneous settling or filtration with or without added pectic enzymes) did not affect the concentration of free terpenols in Falanghina musts and wines, but the glycosylated precursors decreased. The two clarification treatments analyzed in the current study affected the concentrations of those compounds responsible for the varietal aroma (terpenes and norisoprenoids) in individual Albariño or Treixadura wines. In general, the concentrations of these varietal aroma compounds decreased in wines obtained from the flotation technique, but these concentrations are so low in both varieties that they never exceed their corresponding perception thresholds, so that these changes will not be perceived at the sensory level. This suggests that using these techniques, instead of pectolytic enzymes or fining agents, such as bentonite, which are known for altering strongly wine varietal aroma [26,34], can provide a satisfactory elimination of those particles causing turbidity, while maintaining the wine typicity. This is extremely relevant for terpenic varieties such as Albariño, which usually presents high concentrations of linalool [20,23]. In contrast, Treixadura wines do not have a marked terpenic character [21,24], and the effect that fining agents could have on their varietal character would be less significant.

Contents of C6 alcohols were, in general, significantly higher in wines obtained from musts clarified by flotation for both varieties. Several authors reported that the highest mass of grape solids in the must causes an increase in this family of volatile compounds [31,34,35], but remained unchanged in Malvar white wine whose must was clarified by cold settling or by tangential-flow membrane filtration [39]. These alcohols are formed through the action of lypoxygenases from linoleic and linolenic acids during grape processing and, perhaps, enzymatic treatment after clarification favored the liberation of these precursors [40].

The other families of volatile compounds showed an undefined behavior neither linked to the clarification technique, nor to the variety, and the synthesis of some molecules may depend on the must turbidity, initial assimilable nitrogen content, lipid concentration, etc. [41], even from the same clarification technique, whereas contradictory results may be obtained according to the variety [35,37]. It must also be taken into account that the grapes of each variety come from different areas in each Denomination of Origin (DO), with different climates, different degrees of ripeness, etc., and that each winery wants a different type of wine. Furthermore, the different yeasts used in winemaking can modulate the release of volatile compounds differently depending on whether they are *Saccharomyces cerevisiae* yeasts [42], non-*Saccharomyces* [43], or a combination of both [44]. Although each winery used the same yeast for the alcoholic fermentation of the must clarified by both technologies, grape solid particles can affect yeast performances that drive alcoholic fermentation, promoting yeast cell growth, fermentation kinetics, and nitrogen assimilation, with a corresponding impact on wine aroma [41].

However, if we consider the average behavior of each wine variety, the current study proved that the effects of clarification by flotation on the volatiles are minimal in Albariño and Treixadura wines. In general, flotation only increased significantly the concentrations of 1-hexanol, octanoic acid and furfural in Albariño wines, whereas it increased the concentration of benzyl alcohol in Treixadura wines. In the case of Albariño wines, the volatiles that increased in concentration following the flotation technique provide herbaceous (1-hexanol), rancid (octanoic acid), and toasted (furfural) notes to wine aroma. The increase in concentrations of C6 compounds, such as 1-hexanol, could be negative from the sensorial point of view, because these compounds contribute negative nuances when they are present in high concentrations [45]. However, the concentrations in which they have been detected in the samples of the current study were lower than their corresponding odor thresholds [46,47]. In contrast, octanoic acid reached its odor threshold (10 mg/L) in the wines from the flotation technique and can have an incidence on the aroma of the resulting wine. A lower must turbidity allows increasing the concentrations of short-chain (C6, C8, C10) fatty acids, likely released as intermediate metabolites of the long-chain fatty acid synthesis [35]. Furfural content was higher in wines obtained from static settling in both varieties, mainly in Albariño wines, in agreement with the lower concentrations in Italian Riesling icewines obtained from musts clarified by membrane filtration or centrifugation in comparison with must without any treatment [34].

In the case of Treixadura wines, clarification of musts by flotation significantly increased the concentration of benzyl alcohol. This compound is a varietal alcohol and can exist as free or as glycoside in Albariño and Treixadura grapes [48] and, therefore, can be released from bound forms through the action of the enzyme clarification [49], being predominant in Treixadura variety [20]. Benzyl alcohol gives a pleasant aroma to blackberry and fruity [46]; however, the concentrations observed in the wines, from 31 to 206 μg/L in Albariño wines and between 21 and 131 μg/L in Treixadura wines, were below its odor threshold (200 mg/L, [50]) and, consequently, it would not cause a significant change on wine aroma.

Finally, it must be highlighted that the nonvolatile matrix exerts a powerful impact on wine aroma perception, similar to that of the volatile composition [51,52], and, since the wines of the current study did not present significant differences in alcohol content, the marks given by the panelists were similar between clarification techniques. However, panelists tended to give higher scores to wines coming from the flotation technique in the case of both varieties, although these scores were not significantly different. In the current study, the mouthfeel attributes of the wines from the flotation technique tended to a greater quality and this could have positively affected their global scores, as previously reported for Cabernet Sauvignon wines [53].

## 4. Materials and Methods

### 4.1. Wine Samples

Albariño (5) and Treixadura (4) wines used in the current study were elaborated at industrial scale by several wineries from the Rías Baixas and Ribeiro Denomination of Origin (DO), respectively, employing their standard winemaking protocols, except for the clarification process of the musts, which is the factor studied in the current work. After grape pressing, the must was divided into two batches. In half of the samples Novoclair speed (Lamothe-Abiet, Bordeaux, France) pectolytic enzyme was added at 2 g/hL to clarification at 12 °C following the traditional static clarification process, while in the other half Rapidase flotation (DSM Food Specialties, Seclin, France) enzyme (2 mL/hL) was added and then submitted to a clarification by flotation at 14–18 °C by using an Enolmix equipment (Enartis, La Rioja, Spain) with food-grade nitrogen as a gas. In the first case, the process lasts for 12–60 h or 24–48 h, while in the second case the process takes 3–12 h or 4–12 h for Albariño and Treixadura juices, respectively. Then, musts from the same winery were fermented at 16–20 °C in both cases under the same enological conditions (yeast, temperature, etc.). Each winery used the same yeast to ferment the clarified musts using both technologies, but the yeasts differed between wineries: Zymaflore V1 (Laffort, Bordeaux, France), Excellence FW (Lamothe-Abiet, Bordeaux, France), Lalvin QA-23 (Lallemand Inc., Montréal, Canada), Fermivin LVCB (DSM Food Specialties, Seclin, France), and Viniferm Elegancia (Agrovin, Ciudad Real, Spain). Wine samples were collected in 0.75 L bottles directly from each tank and were kept refrigerated (4 °C) until analysis.

### 4.2. Analytical Methods

Basic parameters of wines (including alcohol content and pH, among others) were determined according to the official methods [54]. Analytical determinations in the wines were carried out in triplicate five months after bottling.

Methanol and higher alcohols were determined in triplicate. As internal standard, 1 mL of 4-methyl-2-pentanol (1 g/L) was added to 5 mL of wine. Then, 2 mL of this mixture were injected directly into a Hewlett Packard 5890 gas chromatograph equipped with a flame ionization detector (FID) using an HP-Innowax capillary column (60 m × 0.25 mm i.d.; film thickness 0.25 μm) according to the method described by Bertrand and Ribéreau-Gayon [55].

The remainder of the volatile compounds were extracted as described by Armada et al. [15]. Briefly, a wine sample of 100 mL containing 2 mL of 3-octanol (20 mg/L) and 2 mL of 3,4-dimethyl-phenol (100 mg/L) as internal standards was extracted three times (10, 5, and 5 mL) with dichloromethane (Merck, Darmstadt, Germany). Then, the organic extract was dried with sodium sulfate and concentrated to 0.5 mL under nitrogen, and 3 mL was injected in triplicate in splitless mode (purge time, 30 s; purge rate 70) in a Hewlett Packard HP 5890-I gas chromatograph coupled to a Hewlett Packard 5970 mass spectrometer (Agilent Technologies, Palo Alto, CA, USA). Spectra were recorded in electron impact mode (ionization energy, 70 eV; source temperature, 250 °C), using an HP-Innowax column (60 × 0.25 mm i.d.; film thickness 0.25 μm). The carrier gas was helium (18 psi). The temperature program was isothermal at 45 °C for 1 min, then 3 °C/min to 230 °C with a final isotherm of 25 min. The acquisition was made in scanning mode (mass range, 30–300 amu; 1.9 spectra/s).

Volatile compounds were identified by comparing their mass spectra (MS Chemstation Wiley 7N library) and their retention times with those of the commercial standards from Merck (Darmstadt, Germany), Fluka (Buchs, Switzerland) or Sigma-Aldrich (Steinheim, Germany). For obtaining the calibration curves, five known amounts of the analytes were subjected to the same liquid–liquid extraction as that for the wine samples, and the quantification was carried out by the interpolation of relative peak areas with respect to the response of internal standards. Sulfur compounds (methionol and thiazole) for which pure compounds were not available were referred as a function of the normalized area (as %) respect to the internal standard (3-octanol). Each wine sample was analyzed in triplicate.

### 4.3. Sensory Evaluation

The wine sensory assessment was carried out approximately 15 days after the performance of the gas chromatographic determinations. The panel consisted of nine professional enologists (25–50 years of age, 22% females and 78% males), most of them from the wineries that supplied the samples. All wines were tasted in the same session, but the sessions were not replicated due to the availability of the tasting panel. The wines were served in standard tasting glasses coded with three random numbers and covered with a watch-glass to minimize the loss of volatile compounds. Testing temperature was 10 °C and room temperature was 20–22 °C.

A scorecard including general descriptors for odor and mouthfeel was given to the judges. These descriptors included the frankness and positive aromatic intensity for the olfactory stage; and frankness, positive intensity and persistence for the mouthfeel stage. Moreover, the judges were asked to give a global score for the aroma and the taste of the wines. The descriptors were scored on a scale from 0 to 9. In addition, judges were asked to provide a mark for the overall quality of the wine.

### 4.4. Statistical Analysis

Significant differences between clarification techniques (pre-fermentation) for the average concentrations of each volatile compound were assessed using paired t tests. Normality and homoscedasticity assumptions were checked with Shapiro–Wilk and Bartlett tests, respectively. Principal component analysis (PCA) was applied to discriminate among the sums of families of volatile compounds in the samples according to the clarification technique. Statistical analysis was conducted using the R environment v. 3.6.2 (The R Foundation, Vienna, Austria) [56].

## 5. Conclusions

The current study provided a preliminary assessment, at an industrial scale, of the effect of two clarification techniques on the volatile composition of Albariño and Treixadura wines. Most volatile compounds in the analyzed wines showed similar concentrations independently of the clarification method employed. However, flotation increased the concentrations of 1-hexanol, octanoic acid and furfural in Albariño wines, and that of benzyl alcohol in Treixadura wines. Sensory evaluation showed a slight trend to high scores of wines from the flotation technique in both Albariño and Treixadura varieties. Therefore, the current study suggests that must clarification through flotation has advantages over the static settling: it saves time and, consequently, decreases the costs, does not change the chemical basic parameters (alcoholic content, pH, etc.), does not reduce the concentration of relevant volatile compounds, and experts tend to value more the global quality of wines coming from the flotation technique; however, further research is needed to evaluate the combination of these physical methods with fining agents and assess their effects on wine flavor chemistry.

## Figures and Tables

**Table 1:** General parameters of the Albariño and Treixadura wines studied (individual and average ± standard error) as a function of the must clarification treatment (S: static settling; F: flotation).

Sample	Alcohol (% vol.)		pH		Total Acidity (g Tartaric Acid/L)		Volatile Acidity (g Acetic Acid/L)	
	S	F	S	F	S	F	S	F
**Albariño wines**								
A1	12.4 ± 0.2	12.4 ± 0.1	*3.32 ± 0.02*	*3.43 ± 0.01*	*7.80 ± 0.14*	*7.10 ± 0.10*	*0.40 ± 0.02*	*0.20 ± 0.02*
A2	*13.3 ± 0.2*	*12.1 ± 0.2*	*3.36 ± 0.01*	*3.03 ± 0.01*	*7.60 ± 0.10*	*10.42 ± 0.13*	0.30 ± 0.01	0.30 ± 0.02
A3	12.5 ± 0.2	12.3 ± 0.1	*3.10 ± 0.02*	*3.26 ± 0.02*	*7.34 ± 0.11*	*6.12 ± 0.10*	0.38 ± 0.01	0.41 ± 0.02
A4	12.1 ± 0.1	12.0 ± 0.2	*3.56 ± 0.02*	*3.37 ± 0.01*	*5.80 ± 0.10*	*6.10 ± 0.12*	*0.40 ± 0.01*	*0.20 ± 0.03*
A5	12.3 ± 0.3	12.2 ± 0.1	*3.51 ± 0.01*	*3.40 ± 0.02*	*7.60 ± 0.14*	*6.30 ± 0.13*	0.30 ± 0.02	0.30 ± 0.01
**Average**	12.5 ± 0.1	12.2 ± 0.1	3.37 ± 0.06	3.30 ± 0.05	7.23 ± 0.30	7.21 ± 0.60	0.36 ± 0.02	0.28 ± 0.03
***p*-value**	0.223		0.477		0.976		0.226	
**Treixadura wines**								
T1	12.7 ± 0.3	12.4 ± 0.2	*3.47 ± 0.02*	*3.58 ± 0.03*	*5.15 ± 0.12*	*4.70 ± 0.11*	*0.27 ± 0.02*	*0.20 ± 0.01*
T2	12.9 ± 0.2	13.0 ± 0.1	*3.26 ± 0.01*	*3.45 ± 0.02*	*6.12 ± 0.14*	*5.40 ± 0.10*	0.32 ± 0.02	0.28 ± 0.03
T3	13.5 ± 0.2	13.0 ± 0.3	3.30 ± 0.01	3.30 ± 0.01	*4.95 ± 0.10*	*5.20 ± 0.13*	0.36 ± 0.01	0.36 ± 0.02
T4	*13.3 ± 0.2*	*12.7 ± 0.2*	3.51 ± 0.02	3.49 ± 0.02	*5.20 ± 0.11*	*4.74 ± 0.12*	0.43 ± 0.01	0.43 ± 0.02
**Average**	13.1 ± 0.2	12.8 ± 0.1	3.39 ± 0.03	3.46 ± 0.05	5.36 ± 0.10	5.01 ± 0.10	0.35 ± 0.03	0.32 ± 0.05
***p*-value**	0.142		0.247		0.213		0.182	

For each pair of wines (S, F), significant differences at the 95% confidence level are shown in italics.

**Table 2:** Volatile composition (average ± standard deviation, *n* = 3) of Albariño wines from the Rías Baixas DO after clarification of the musts by static settling (S) or flotation (F).

Compound	A1-S	A1-F	A2-S	A2-F	A3-S	A3-F	A4-S	A4-F	A5-S	A5-F
**Terpenes and norisoprenoids (μg/L)**										
Linalool	*54.8 ± 0.5*	*37.3 ± 0.8*	36.2 ± 3.4	36.2 ± 0.1	34.8 ± 0.7	34.2 ± 0.9	12.4 ± 0.1	11.5 ± 0.3	*7.02 ± 0.08*	*14.3 ± 0.2*
Terpinen-4-ol	*3.04 ± 0.19*	*n.d.*	*2.23 ± 0.09*	*n.d.*	*2.17 ± 0.17*	*n.d*	*0.68 ± 0.04*	*n.d.*	n.d	n.d.
α-terpineol	*6.70 ± 0.27*	*4.23 ± 0.02*	*4.41 ± 0.04*	*n.d.*	*4.90 ± 0.03*	*2.94 ± 0.04*	*1.33 ± 0.04*	*4.41 ± 0.18*	*1.16 ± 0.10*	*1.71 ± 0.03*
Theaspirane A	*12.1 ± 0.1*	*7.70 ± 0.24*	*17.4 ± 1.6*	*19.0 ± 0.1*	*4.76 ± 0.33*	*15.4 ± 0.0*	n.d	n.d.	n.d	n.d.
β-damascenone	2.00 ± 0.20	2.00 ± 0.50	2.00 ± 0.30	2.30 ± 0.10	1.00 ± 0.30	1.00 ± 0.40	1.00 ± 0.50	1.00 ± 0.40	1.00 ± 0.40	2.00 ± 0.10
β-ionone	*55.5 ± 0.3*	*43.8 ± 0.2*	*6.68 ± 0.01*	*7.09 ± 0.61*	*4.91 ± 0.39*	*15.7 ± 0.3*	n.d.	n.d.	n.d.	n.d.
**C6 compounds (mg/L)**										
1-hexanol	*0.856 ± 0.003*	*1.18 ± 0.00*	*0.858 ± 0.003*	*0.864 ± 0.002*	*0.401 ± 0.002*	*0.650 ± 0.061*	*0.233 ± 0.001*	*0.314 ± 0.003*	*0.268 ± 0.002*	*0.592 ± 0.021*
*trans*-3-hexen-1-ol	*0.033 ± 0.000*	*0.028 ± 0.000*	*0.017 ± 0.000*	*0.018 ± 0.000*	*0.011 ± 0.000*	*0.007 ± 0.000*	*0.003 ± 0.000*	*0.007 ± 0.000*	*0.004 ± 0.000*	*0.016 ± 0.000*
*cis*-3-hexen-1-ol	*0.048 ± 0.000*	*0.070 ± 0.000*	*0.028 ± 0.000*	*0.029 ± 0.000*	*0.014 ± 0.000*	*0.011 ± 0.000*	*0.011 ± 0.000*	*0.014 ± 0.000*	*0.014 ± 0.000*	*0.033 ± 0.000*
*trans*-2-hexen-1-ol	0.002 ± 0.000	0.003 ± 0.000	0.004 ± 0.000	0.004 ± 0.000	0.001 ± 0.000	0.001 ± 0.000	0.001 ± 0.000	0.001 ± 0.000	*0.001* ± 0.000	*0.004* ± 0.000
**Alcohols (mg/L)**										
Methanol	*49.6 ± 0.6*	*36.2 ± 1.3*	23.9 ± 0.2	24.4 ± 0.6	*28.1 ± 0.9*	*33.7 ± 1.4*	*30.8 ± 0.3*	*89.7 ± 3.6*	*37.6 ± 0.3*	*33.6 ± 0.6*
1-propanol	22.5 ± 0.3	23.5 ± 0.8	15.8 ± 0.9	15.9 ± 0.8	*12.8 ± 0.5*	*22.7 ± 0.6*	*19.0 ± 0.9*	*8.58 ± 0.8*	*18.7 ± 0.5*	*16.8 ± 0.6*
Isobutanol	*23.0 ± 0.8*	*12.2 ± 0.5*	*12.9 ± 0.4*	*15.8 ± 0.6*	*16.2 ± 0.8*	*10.5 ± 0.5*	*6.88 ± 0.21*	*11.2 ± 0.5*	8.98 ± 0.83	7.40 ± 0.42
1-butanol	*0.550 ± 0.002*	*0.525 ± 0.002*	*0.550 ± 0.004*	*0.578 ± 0.003*	*0.615 ± 0.005*	*0.468 ± 0.002*	*0.146 ± 0.002*	*0.521 ± 0.003*	*0.107 ± 0.001*	*0.910 ± 0.005*
Isoamyl alcohol	*105 ± 0.2*	*83.6 ± 0.2*	*84.9 ± 0.1*	*90.4 ± 0.2*	*96.4 ± 0.1*	*102 ± 0.3*	*33.1 ± 0.1*	*37.3 ± 0.1*	*34.6 ± 0.1*	*66.0 ± 0.1*
3-methyl-1-pentanol	*0.191 ± 0.001*	*n.d.*	0.032 ± 0.003	0.039 ± 0.009	0.166 ± 0.038	0.086 ± 0.065	*0.021 ± 0.000*	*0.517 ± 0.051*	*0.106 ± 0.008*	*0.049 ± 0.024*
Furfuryl alcohol	*0.235 ± 0.001*	*0.089 ± 0.001*	*0.412 ± 0.004*	*0.475 ± 0.003*	*0.128 ± 0.008*	*0.100 ± 0.001*	n.d.	n.d.	n.d.	n.d.
Benzyl alcohol	*0.057 ± 0.002*	*0.075 ± 0.002*	*0.156 ± 0.001*	*0.206 ± 0.002*	*0.089 ± 0.000*	*0.065 ± 0.004*	*0.043 ± 0.000*	*0.031 ± 0.001*	*0.069 ± 0.000*	*0.149 ± 0.002*
2-phenyl-ethanol	*18.6 ± 0.1*	*12.9 ± 0.1*	*9.24 ± 0.1*	*10.1 ± 0.1*	*29.0 ± 0.1*	*13.4 ± 0.1*	*12.9 ± 0.1*	*20.0 ± 0.1*	*23.6 ± 0.1*	*10.4 ± 0.1*
**Acetates of higher alcohols (mg/L)**										
Isobutyl acetate	0.062 ± 0.004	0.058 ± 0.001	0.055 ± 0.001	0.048 ± 0.005	*0.035 ± 0.001*	*0.054 ± 0.008*	*0.081 ± 0.003*	*0.068 ± 0.000*	*0.004 ± 0.000*	*0.094 ± 0.004*
Butyl acetate	*0.142 ± 0.010*	*0.037 ± 0.013*	*0.065 ± 0.008*	*0.250 ± 0.008*	*0.098 ± 0.011*	*0.007 ± 0.001*	*0.089 ± 0.006*	*0.015 ± 0.004*	*0.033 ± 0.003*	*0.103 ± 0.015*
Isoamyl acetate	*2.00 ± 0.00*	*2.66 ± 0.01*	1.59 ± 0.00	1.59 ± 0.00	*1.97 ± 0.00*	*1.29 ± 0.00*	*2.25 ± 0.00*	*0.966 ± 0.001*	*1.15 ± 0.00*	*1.95 ± 0.00*
Hexyl acetate	*0.323 ± 0.000*	*0.352 ± 0.002*	0.121 ± 0.002	0.120 ± 0.002	*0.082 ± 0.000*	*0.059 ± 0.002*	*0.158 ± 0.000*	*0.073 ± 0.000*	*0.100 ± 0.002*	*0.167 ± 0.002*
*cis*-3-hexenyl acetate	*0.006 ± 0.001*	*0.014 ± 0.001*	0.005 ± 0.000	0.004 ± 0.000	*0.001 ± 0.000*	*0.004 ± 0.000*	*0.009 ± 0.000*	*0.006 ± 0.000*	*0.017 ± 0.001*	*0.006 ± 0.000*
2-phenylethyl acetate	*0.008 ± 0.000*	*0.005 ± 0.001*	n.d.	n.d.	0.002 ± 0.000	0.001 ± 0.000	0.001 ± 0.000	0.002 ± 0.000	*0.004* ± 0.000	*n.d.*
**Ethyl esters (mg/L)**										
Ethyl 2-methyl-butyrate	n.d.	n.d.	0.008 ± 0.000	0.009 ± 0.000	*0.007 ± 0.000*	*0.022 ± 0.001*	*0.002 ± 0.000*	*n.d.*	n.d.	n.d.
Ethyl isovalerate	*0.015 ± 0.000*	*0.009 ± 0.000*	*0.010 ± 0.000*	*0.012 ± 0.000*	*0.010 ± 0.000*	*0.009 ± 0.000*	0.002 ± 0.000	0.002 ± 0.000	*0.002 ± 0.000*	*0.006 ± 0.000*
Ethyl hexanoate	*0.726 ± 0.001*	*0.704 ± 0.002*	0.587 ± 0.000	0.588 ± 0.001	*0.792 ± 0.001*	*0.981 ± 0.003*	*0.232 ± 0.000*	*0.313 ± 0.000*	*0.203 ± 0.000*	*0.726 ± 0.002*
Ethyl octanoate	*1.22 ± 0.00*	*1.31 ± 0.00*	*1.01 ± 0.00*	*1.04 ± 0.00*	*1.41 ± 0.00*	*1.69 ± 0.00*	0.366 ± 0.001	0.366 ± 0.001	*0.355 ± 0.003*	*0.831 ± 0.002*
Ethyl 3-hydroxybutyrate	*0.192 ± 0.000*	*0.110 ± 0.000*	0.125 ± 0.003	0.130 ± 0.001	0.106 ± 0.001	0.103 ± 0.001	*0.055 ± 0.000*	*0.041 ± 0.000*	*0.056 ± 0.004*	*0.071 ± 0.001*
Ethyl decanoate	1.95 ± 0.00	1.96 ± 0.01	*1.93 ± 0.00*	*2.01 ± 0.00*	*2.16 ± 0.00*	*2.80 ± 0.00*	*0.600 ± 0.002*	*0.728 ± 0.001*	*0.661 ± 0.003*	*1.76 ± 0.00*
Isoamyl octanoate	*0.004 ± 0.000*	*0.007 ± 0.000*	*0.005 ± 0.000*	*n.d.*	0.006 ± 0.000	0.005 ± 0.000	*0.006 ± 0.000*	*0.002 ± 0.000*	0.004 ± 0.000	0.003 ± 0.000
Ethyl lactate	*9.12 ± 0.02*	*3.83 ± 0.01*	5.71 ± 0.01	5.70 ± 0.02	*6.84 ± 0.07*	*4.21 ± 0.01*	*1.18 ± 0.00*	*10.4 ± 0.1*	*1.11 ± 0.00*	*31.7 ± 0.1*
Diethyl succinate	*0.620 ± 0.001*	*0.239 ± 0.000*	*0.408 ± 0.000*	*0.434 ± 0.001*	*0.466 ± 0.001*	*0.392 ± 0.001*	*0.046 ± 0.000*	*0.062 ± 0.000*	*0.121 ± 0.000*	*0.094 ± 0.001*
Monoethyl succinate	*38.1 ± 0.1*	*20.9 ± 0.1*	*22.5 ± 0.1*	*24.6 ± 0.1*	*45.2 ± 0.3*	*53.1 ± 0.3*	*4.15 ± 0.1*	*9.98 ± 0.1*	*15.4 ± 0.2*	*23.0 ± 0.1*
**Fatty acids (mg/L)**										
Isobutyric acid	*2.10 ± 0.03*	*1.39 ± 0.02*	2.24 ± 0.02	2.27 ± 0.01	*1.64 ± 0.03*	*1.39 ± 0.01*	*0.436 ± 0.007*	*1.55 ± 0.04*	*0.396 ± 0.015*	*1.80 ± 0.08*
Butyric acid	*3.81 ± 0.03*	*3.32 ± 0.03*	*3.38 ± 0.01*	*3.49 ± 0.01*	*3.15 ± 0.01*	*4.10 ± 0.02*	*0.955 ± 0.002*	*1.19 ± 0.00*	*1.02 ± 0.02*	*2.31 ± 0.00*
Isovaleric acid	*1.05 ± 0.00*	*1.01 ± 0.00*	*1.18 ± 0.00*	*1.22 ± 0.00*	*0.918 ± 0.002*	*0.823 ± 0.004*	*0.276 ± 0.000*	*0.336 ± 0.000*	*0.344 ± 0.000*	*0.846 ± 0.004*
Valeric acid	*0.750 ± 0.013*	*1.04 ± 0.00*	*0.481 ± 0.003*	*0.503 ± 0.004*	*0.828 ± 0.004*	*0.534 ± 0.005*	*0.637 ± 0.013*	*0.253 ± 0.001*	*0.540 ± 0.005*	*0.492 ± 0.010*
Hexanoic acid	*7.44 ± 0.02*	*7.74 ± 0.02*	*6.04 ± 0.01*	*6.29 ± 0.01*	*7.63 ± 0.01*	*10.8 ± 0.1*	*2.05 ± 0.01*	*3.41 ± 0.00*	*2.16 ± 0.00*	*6.59 ± 0.01*
Octanoic acid	*10.8 ± 0.1*	*12.5 ± 0.1*	*8.91 ± 0.10*	*9.70 ± 0.02*	*12.0 ± 0.1*	*16.6 ± 0.1*	*3.32 ± 0.01*	*5.40 ± 0.01*	*3.50 ± 0.00*	*7.97 ± 0.92*
Decanoic acid	*4.32 ± 0.00*	*4.59 ± 0.01*	*3.57 ± 0.01*	*3.81 ± 0.04*	*3.86 ± 0.01*	*4.30 ± 0.02*	*1.30 ± 0.00*	*1.65 ± 0.00*	*1.35 ± 0.00*	*4.17 ± 0.00*
Lauric acid	*0.488 ± 0.011*	*0.367 ± 0.006*	*0.394 ± 0.022*	*0.369 ± 0.001*	*0.477 ± 0.014*	*0.268 ± 0.007*	0.090 ± 0.003	0.096 ± 0.002	*0.106 ± 0.001*	*0.233 ± 0.002*
**Volatile phenols (mg/L)**										
Phenyl acetaldehyde	*0.008 ± 0.000*	*0.007 ± 0.000*	0.008 ± 0.000	0.008 ± 0.000	0.007 ± 0.000	0.008 ± 0.000	0.003 ± 0.000	0.004 ± 0.000	*0.002 ± 0.000*	*0.012 ± 0.001*
Guaiacol	*0.041 ± 0.000*	*0.006 ± 0.000*	0.005 ± 0.000	0.005 ± 0.000	*0.027 ± 0.000*	*0.061 ± 0.000*	*0.005 ± 0.000*	*0.013 ± 0.000*	n.d.	n.d.
4-ethyl-guaiacol	*0.310 ± 0.037*	*0.215 ± 0.028*	*0.766 ± 0.038*	*3.91 ± 0.04*	*1.29 ± 0.03*	*n.d*	*0.363 ± 0.010*	*0.146 ± 0.012*	*0.021 ± 0.006*	*0.243 ± 0.012*
4-vinyl-guaiacol	*1.62 ± 0.00*	*0.837 ± 0.015*	1.69 ± 0.03	1.77 ± 0.05	*0.854 ± 0.006*	*0.747 ± 0.012*	*0.408 ± 0.010*	*0.220 ± 0.001*	*0.362 ± 0.002*	*0.464 ± 0.012*
Isoeugenol	*0.011 ± 0.000*	*0.005 ± 0.000*	0.009 ± 0.000	0.009 ± 0.001	0.002 ± 0.000	0.002 ± 0.000	n.d	n.d	n.d.	n.d.
4-vinyl-phenol	*7.45 ± 0.27*	*1.63 ± 0.07*	*5.54 ± 0.07*	*6.08 ± 0.31*	2.52 ± 0.03	2.14 ± 0.38	*0.400 ± 0.002*	*0.526 ± 0.057*	*0.442 ± 0.021*	*0.729 ± 0.023*
Vanillin	0.023 ± 0.002	0.024 ± 0.003	*n.d.*	*0.009 ± 0.001*	n.d	n.d	n.d.	n.d.	*n.d.*	*0.019 ± 0.01*
Ethyl vanillate	*0.131 ± 0.000*	*0.174 ± 0.001*	*0.126 ± 0.001*	*0.064 ± 0.001*	*0.057 ± 0.000*	*0.045 ± 0.000*	*0.028 ± 0.000*	*0.012 ± 0.001*	*0.013 ± 0.000*	*0.036 ± 0.001*
**Carbonyl compounds (mg/L)**										
Acetoine	*2.42 ± 0.03*	*1.70 ± 0.01*	*2.00 ± 0.00*	*2.05 ± 0.00*	*1.39 ± 0.00*	*1.70 ± 0.00*	*0.618 ± 0.002*	*7.80 ± 0.01*	*0.562 ± 0.003*	*17.6 ± 0.0*
Furfural	*0.153 ± 0.003*	*0.052 ± 0.005*	*0.074 ± 0.003*	*0.027 ± 0.006*	*n.d.*	*0.156 ± 0.030*	*n.d.*	*6.38 ± 0.57*	*0.126 ± 0.004*	*0.063 ± 0.036*
Benzaldehyde	*0.026 ± 0.000*	*0.009 ± 0.000*	*0.034 ± 0.001*	*0.039 ± 0.001*	0.016 ± 0.001	0.016 ± 0.000	0.002 ± 0.000	0.002 ± 0.000	0.007 ± 0.001	0.008 ± 0.001
**Sulfur compounds (% normalized area)**										
Methionol	*32.04 ± 0.29*	*26.14 ± 0.10*	*26.44 ± 0.14*	*27.56 ± 0.09*	*53.49 ± 0.67*	*62.92 ± 0.18*	*16.63 ± 0.09*	*23.25 ± 0.15*	*23.92 ± 0.18*	*29.43 ± 0.29*
Thiazole	*86.44 ± 0.59*	*55.36 ± 0.69*	*76.41 ± 0.67*	*80.66 ± 0.12*	*87.02 ± 0.38*	*91.83 ± 0.38*	*15.14 ± 0.05*	*n.d.*	*31.93 ± 0.15*	*27.11 ± 0.07*

n.d.: not detected. For each pair of wines (S, F), significant differences at the 95% confidence level are shown in italics.

**Table 3:** Volatile composition (average *±* standard deviation, *n* = 3) of Treixadura wines from the Ribeiro DO after clarification of the musts by static settling (S) or flotation (F).

Compound	T1-S	T1-F	T2-S	T2-F	T3-S	T3-F	T4-S	T4-F
**Terpenes and norisoprenoids (μg/L)**								
Linalool	n.d.	n.d.	n.d.	n.d.	*7.42 ± 0.15*	*8.68 ± 0.07*	n.d.	n.d.
Terpinen-4-ol	n.d.	n.d.	*3.57* ± 0.15	*2.57* ± 0.07	*2.08 ± 0.10*	*n.d.*	n.d.	n.d.
α-terpineol	n.d.	n.d.	n.d.	n.d.	*1.71 ± 0.04*	*1.58 ± 0.01*	n.d.	n.d.
Theaspirane A	n.d.	n.d.	n.d.	n.d.	*6.91 ± 0.03*	*n.d.*	n.d.	n.d.
**C6 compounds (mg/L)**								
1-hexanol	*0.524 ± 0.002*	*0.781 ± 0.005*	*0.521 ± 0.004*	*0.693 ± 0.006*	*0.693 ± 0.030*	*0.523 ± 0.005*	*0.778 ± 0.011*	*0.925 ± 0.013*
*trans*-3-hexen-1-ol	*0.009* ± 0.000	*0.017* ± 0.001	*0.041* ± 0.001	*0.032* ± 0.002	0.016 ± 0.001	0.011 ± 0.000	0.015 ± 0.000	0.022 ± 0.000
*cis*-3-hexen-1-ol	*0.024 ± 0.000*	*0.027 ± 0.000*	*0.022 ± 0.000*	*0.018 ± 0.001*	0.022 ± 0.001	0.024 ± 0.001	*0.027 ± 0.001*	*0.034 ± 0.001*
*trans*-2-hexen-1-ol	n.d.	n.d.	0.003 ± 0.000	0.003 ± 0.000	0.003 ± 0.000	0.003 ± 0.000	0.002 ± 0.000	0.002 ± 0.000
**Alcohols (mg/L)**								
Methanol	*24.2 ± 0.0*	*60.7 ± 1.3*	*37.0 ± 1.0*	*41.2 ± 0.6*	*29.5 ± 0.3*	*118 ± 1*	*28.5 ± 0.4*	*36.0 ± 2.3*
1-propanol	*20.3 ± 0.4*	*27.7 ± 0.7*	*18.0 ± 0.1*	*15.8 ± 1.2*	18.6 ± 0.1	17.6 ± 0.8	*36.1 ± 0.2*	*12.2 ± 0.4*
Isobutanol	*10.0 ± 0.1*	*10.8 ± 0.1*	*22.4 ± 0.1*	*18.9 ± 0.1*	*13.3 ± 0.0*	*8.49 ± 0.02*	*7.24 ± 0.02*	*9.07 ± 0.08*
1-butanol	*0.553 ± 0.005*	*0.689 ± 0.006*	*0.461 ± 0.003*	*0.283 ± 0.010*	*0.441 ± 0.001*	*3.12 ± 0.01*	*0.616 ± 0.017*	*0.545 ± 0.016*
Isoamyl alcohol	*91.7 ± 0.7*	*111 ± 0.7*	*105 ± 0.2*	*131 ± 0.2*	*83.1 ± 0.4*	*78.2 ± 0.2*	*82.0 ± 0.1*	*86.3 ± 0.2*
3-methyl-1-pentanol	*0.069 ± 0.007*	*0.166 ± 0.098*	*1.04 ± 0.03*	*0.143 ± 0.013*	*0.083 ± 0.014*	*0.487 ± 0.093*	0.106 ± 0.048	0.132 ± 0.027
Furfuryl alcohol	n.d.	n.d.	*143 ± 2.4*	*n.d.*	*167 ± 19*	*103 ± 0.9*	n.d.	n.d.
Benzyl alcohol	*0.071 ± 0.000*	*0.191 ± 0.002*	*0.050 ± 0.000*	*0.129 ± 0.004*	*0.045 ± 0.000*	*0.131 ± 0.006*	*0.021 ± 0.001*	*0.035 ± 0.001*
2-phenyl-ethanol	*10.1 ± 0.2*	*17.7 ± 0.5*	*32.2 ± 0.8*	*15.2 ± 0.3*	*11.5 ± 0.1*	*26.7 ± 1.5*	*14.6 ± 0.2*	*12.7 ± 0.1*
**Acetates of higher alcohols (mg/L)**								
Isobutyl acetate	*0.016 ± 0.000*	*0.065 ± 0.012*	0.119 ± 0.003	0.109 ± 0.009	*0.083 ± 0.005*	*0.047 ± 0.001*	*0.036 ± 0.002*	*0.032 ± 0.001*
Isoamyl acetate	*4.75 ± 0.03*	*5.14 ± 0.03*	*6.44 ± 0.01*	*4.28 ± 0.00*	*1.80 ± 0.01*	*3.12 ± 0.01*	*3.83 ± 0.00*	*3.18 ± 0.01*
Hexyl acetate	*0.235 ± 0.001*	*0.326 ± 0.001*	*0.263 ± 0.002*	*0.174 ± 0.000*	*0.141 ± 0.002*	*0.156 ± 0.002*	*0.362 ± 0.001*	*0.337 ± 0.000*
Benzyl acetate	*1.56 ± 0.04*	*2.28 ± 0.02*	*0.179 ± 0.011*	*0.309 ± 0.005*	*0.111 ± 0.001*	*0.220 ± 0.004*	*0.231 ± 0.002*	*0.177 ± 0.000*
*cis*-3-hexenyl acetate	0.006 ± 0.001	0.007 ± 0.000	*0.001 ± 0.000*	*0.010 ± 0.001*	*0.007 ± 0.001*	*0.003 ± 0.000*	*0.009* ± 0.000	*0.008* ± 0.000
2-phenylethyl acetate	*n.d.*	*0.004*± 0.000	*n.d.*	*0.001 ± 0.000*	*0.003 ± 0.000*	*0.001 ± 0.000*	*n.d.*	*0.001 ± 0.000*
**Ethyl esters (mg/L)**								
Ethyl-butyrate	*0.518 ± 0.024*	*0.554 ± 0.080*	*0.536 ± 0.030*	*0.440 ± 0.027*	*0.540 ± 0.013*	*0.388 ± 0.020*	*0.581 ± 0.020*	*0.431 ± 0.003*
Ethyl isovalerate	*5.50 ± 0.06*	*3.90 ± 0.04*	n.d.	n.d.	*n.d.*	*6.85 ± 0.06*	*3.20 ± 0.05*	*3.69 ± 0.07*
Ethyl hexanoate	*0.533 ± 0.005*	*0.574 ± 0.003*	*0.510 ± 0.004*	*0.466 ± 0.026*	0.515 ± 0.022	0.512 ± 0.005	*0.654 ± 0.010*	*0.567 ± 0.002*
Ethyl octanoate	*1.27 ± 0.01*	*1.32 ± 0.01*	*1.03 ± 0.00*	*1.01 ± 0.00*	*1.19 ± 0.00*	*1.22 ± 0.01*	*1.24 ± 0.00*	*1.15 ± 0.00*
Ethyl 3-hydroxybutyrate	*0.141 ± 0.001*	*0.171 ± 0.000*	*0.188 ± 0.001*	*0.108 ± 0.000*	*0.147 ± 0.001*	*0.206 ± 0.001*	*0.120 ± 0.000*	*0.107 ± 0.002*
Ethyl decanoate	*0.268 ± 0.002*	*0.445 ± 0.022*	0.218 ± 0.007	0.238 ± 0.011	0.285 ± 0.028	0.255 ± 0.003	*0.243 ± 0.009*	*0.197 ± 0.007*
Isoamyl octanoate	*1.63 ± 0.01*	*1.60 ± 0.01*	*0.775 ± 0.035*	*0.509 ± 0.024*	*2.03 ± 0.01*	*2.20 ± 0.02*	*0.352 ± 0.011*	*0.391 ± 0.008*
Ethyl lactate	*7.86 ± 0.06*	*2.49 ± 0.02*	*9.79 ± 0.02*	*9.25 ± 0.01*	*36.6 ± 1.4*	*28.7 ± 0.1*	*2.19 ± 0.01*	*1.99 ± 0.02*
Diethyl succinate	*0.096 ± 0.002*	*0.088 ± 0.000*	*n.d.*	*0.179 ± 0.011*	*0.126 ± 0.003*	*0.333 ± 0.002*	n.d.	n.d.
Monoethyl succinate	*14.5 ± 0.2*	*13.6± 0.1*	*16.9 ± 0.2*	*24.6 ± 0.2*	*19.7 ± 0.1*	*27.5 ± 0.1*	*10.0 ± 0.1*	*13.6 ± 0.1*
**Fatty acids (mg/L)**								
Isobutyric acid	*0.143 ± 0.003*	*1.03 ± 0.01*	*1.46 ± 0.03*	*1.79 ± 0.06*	*1.76 ± 0.04*	*1.22 ± 0.03*	0.676 ± 0.011	0.683 ± 0.008
Butyric acid	*3.20 ± 0.03*	*3.47 ± 0.04*	*3.99 ± 0.07*	*3.65 ± 0.07*	*3.62 ± 0.03*	*3.25 ± 0.07*	*3.19 ± 0.01*	*3.30 ± 0.03*
Isovaleric acid	*0.861 ± 0.003*	*0.790 ± 0.001*	*1.19 ± 0.00*	*1.67 ± 0.01*	*0.639 ± 0.018*	*0.805 ± 0.017*	*0.761 ± 0.003*	*0.712 ± 0.004*
Hexanoic acid	*6.52 ± 0.05*	*7.84 ± 0.05*	*6.34 ± 0.04*	*6.73 ± 0.02*	*7.27 ± 0.02*	*6.31 ± 0.02*	*7.22 ± 0.02*	*7.49 ± 0.02*
Octanoic acid	*11.2 ± 0.1*	*13.7 ± 0.1*	9.53 ± 0.02	9.59 ± 0.03	*11.4 ± 0.06*	*9.78 ± 0.02*	*10.9 ± 0.0*	*10.5 ± 0.0*
Decanoic acid	*4.09 ± 0.03*	*4.72 ± 0.04*	*3.34 ± 0.02*	*2.90 ± 0.01*	*3.68 ± 0.02*	*3.98 ± 0.01*	*3.54 ± 0.00*	*3.23 ± 0.01*
Lauric acid	*0.300 ± 0.003*	*0.575 ± 0.011*	*0.275 ± 0.009*	*0.122 ± 0.004*	*0.172 ± 0.004*	*0.279 ± 0.007*	*0.150 ± 0.001*	*0.177 ± 0.003*
**Volatile phenols (mg/L)**								
Phenyl acetaldehyde	n.d.	n.d.	*0.022 ± 0.000*	*0.015 ± 0.002*	*0.006 ± 0.000*	*0.004 ± 0.000*	0.004 ± 0.000	0.004 ± 0.001
β-damascone	0.001 ± 0.000	0.001 ± 0.000	*0.001 ± 0.000*	*n.d.*	0.001 ± 0.000	0.001 ± 0.000	0.001 ± 0.000	0.001 ± 0.000
Guaiacol	*0.061 ± 0.000*	*0.010 ± 0.008*	*0.206 ± 0.001*	*0.158 ± 0.000*	*0.017 ± 0.000*	*0.072 ± 0.000*	*0.014 ± 0.000*	*0.007 ± 0.000*
4-ethyl-guaiacol	*n.d.*	*0.005 ± 0.000*	*20.4 ± 0.2*	*30.4 ± 2.0*	*16.0 ± 0.2*	*14.0 ± 0.3*	14.2 ± 0.1	14.0 ± 0.1
4-vinyl-guaiacol	*0.994 ± 0.016*	*1.06 ± 0.01*	*0.858 ± 0.038*	*0.740 ± 0.017*	*0.585 ± 0.008*	*0.663 ± 0.008*	*1.28 ± 0.01*	*1.05 ± 0.01*
4-vinyl-phenol	*1.89 ± 0.02*	*2.59 ± 0.03*	*1.05 ± 0.01*	*0.263 ± 0.030*	1.59 ± 0.06	1.54 ± 0.01	*1.72 ± 0.05*	*2.04 ± 0.02*
Vanillin	*0.008* ± 0.000	*0.016* ± 0.000	n.d.	n.d.	n.d.	n.d.	n.d.	n.d.
Ethyl vanillate	142 ± 1.0	141 ± 0.4	*15.9 ± 0.3*	*54.7 ± 0.1*	*48.0 ± 0.2*	*39.4 ± 0.5*	*96.9 ± 1.3*	*68.5 ± 0.6*
**Carbonyl compounds (mg/L)**								
Acetoine	*1.70 ± 0.01*	*1.39 ± 0.00*	*4.88 ± 0.01*	*0.875 ± 0.038*	*4.22 ± 0.09*	*6.04 ± 0.154*	*0.862 ± 0.016*	*1.08 ± 0.01*
Furfural	*0.106 ± 0.005*	*0.065 ± 0.014*	*0.064 ± 0.002*	*0.114 ± 0.014*	*0.111 ± 0.010*	*0.190 ± 0.051*	*0.046 ± 0.011*	*n.d.*
Benzaldehyde	*0.014 ± 0.001*	*0.027 ± 0.000*	*0.007 ± 0.000*	*n.d.*	*0.007 ± 0.000*	*0.009 ± 0.000*	*0.003 ± 0.000*	*0.014 ± 0.001*
**Sulfur compounds (% normalized area)**								
Methionol	33.18 ± 0.94	33.80 ± 0.19	*38.86 ± 0.82*	*136.56 ± 1.15*	*55.77 ± 0.20*	*3.32 ± 0.26*	*17.48 ± 0.07*	*23.41 ± 0.11*
Thiazole	*19.91 ± 0.08*	*22.78 ± 0.51*	*7.32 ± 0.35*	*11.24 ± 0.56*	*3.52 ± 0.29*	*44.16 ± 0.06*	n.d.	n.d.

n.d.: not detected. For each pair of wines (S, F), significant differences at the 95% confidence level are shown in italics.

**Table 4:** Average concentrations of volatile compounds (mean ± standard error) in Albariño and Treixadura wines after static settling or flotation clarification of the musts.

	Albariño Wines			Treixadura Wines		
Compound	Static	Flotation	*p*-Value	Static	Flotation	*p*-Value
**Terpenes and norisoprenoids (μg/L)**						
Linalool	29.0 ± 6.9	26.7 ± 4.9	0.596	7.42 ± 0.00	8.68 ± 0.00	--
Terpinen-4-ol	2.03 ± 0.3	n.d.	--	2.83 ± 0.37	2.57 ± 0.00	--
α-terpineol	3.70 ± 0.9	3.30 ± 0.5	0.886	1.72 ± 0.00	1.58 ± 0.00	--
Theaspirane A	11.4 ± 2.0	14.0 ± 1.9	0.616	6.91 ± 0.00	n.d.	--
β-damascenone	1.40 ± 0.25	1.66 ± 0.28	0.251	n.d.	n.d.	--
β-ionone	22.4 ± 9.9	22.2 ± 6.5	0.983	n.d.	n.d.	--
**C6 compounds (mg/L)**						
1-hexanol	0.523 ± 0.119	0.721 ± 0.109	0.039	0.629 ± 0.053	0.731 ± 0.061	0.357
*trans*-3-hexen-1-ol	0.014 ± 0.004	0.015 ± 0.003	0.630	0.020 ± 0.005	0.021 ± 0.003	0.957
*cis*-3-hexen-1-ol	0.023 ± 0.005	0.031 ± 0.007	0.172	0.024 ± 0.001	0.026 ± 0.002	0.444
*trans*-2-hexen-1-ol	0.002 ± 0.000	0.003 ± 0.001	0.242	0.003 ± 0.000	0.003 ± 0.000	0.999
**Alcohols (mg/L)**						
Methanol	34.0 ± 3.4	43.5 ± 8.3	0.496	29.8 ± 1.8	63.9 ± 13.4	0.178
1-propanol	17.8 ± 1.2	17.5 ± 2.0	0.941	23.2 ± 3.2	18.3 ± 2.3	0.516
Isobutanol	13.6 ± 2.1	11.4 ± 0.9	0.481	13.2 ± 2.3	11.8 ± 1.8	0.437
1-butanol	0.39 ± 0.10	0.60 ± 0.06	0.297	0.52 ± 0.03	1.16 ± 0.49	0.417
Isoamyl alcohol	70.7 ± 13.2	75.9 ± 8.7	0.570	90.5 ± 4.0	101 ± 9	0.207
3-methyl-1-pentanol	0.10 ± 0.03	0.18 ± 0.08	0.549	0.32 ± 0.18	0.23 ± 0.06	0.765
Furfuryl alcohol	0.26 ± 0.05	0.22 ± 0.08	0.623	155 ± 6	103 ± 0	--
Benzyl alcohol	0.08 ± 0.01	0.08 ± 0.03	0.310	0.05 ± 0.01	0.12 ± 0.02	0.043
2-phenyl-ethanol	18.7 ± 2.7	18.7 ± 1.2	0.280	17.1 ± 3.8	18.1 ± 2.2	0.899
**Acetates of higher alcohols (mg/L)**						
Isobutyl acetate	0.047 ± 0.010	0.064 ± 0.006	0.422	0.064 ± 0.019	0.063 ± 0.012	0.990
Butyl acetate	0.085 ± 0.018	0.082 ± 0.045	0.960	n.d.	n.d.	--
Isoamyl acetate	1.79 ± 0.15	1.69 ± 0.22	0.813	4.21 ± 0.69	3.93 ± 0.39	0.738
Hexyl acetate	0.157 ± 0.030	0.154 ± 0.038	0.924	0.250 ± 0.031	0.248 ± 0.042	0.961
*cis*-3-hexenyl acetate	0.008 ± 0.002	0.007 ± 0.001	0.813	0.006 ± 0.001	0.007 ± 0.001	0.684
Benzyl acetate	n.d.	n.d.	--	0.521 ± 0.261	0.746 ± 0.383	0.274
2-phenylethyl acetate	0.008 ± 0.001	0.003 ± 0.001	0.478	0.003 ± 0.000	0.002 ± 0.001	--
**Ethyl esters (mg/L)**						
Ethyl 2-methyl-butyrate	0.006 ± 0.001	0.016 ± 0.003	0.458	n.d.	n.d.	-
Ethyl butyrate	n.d.	n.d.	--	0.54 ± 0.01	0.45 ± 0.03	0.132
Ethyl isovalerate	0.008 ± 0.002	0.008 ± 0.001	0.911	4.35 ± 0.58	4.81 ± 0.68	0.686
Ethyl hexanoate	0.508 ± 0.105	0.662 ± 0.076	0.204	0.55 ± 0.03	0.53 ± 0.02	0.459
Ethyl octanoate	0.872 ± 0.183	1.048 ± 0.162	0.123	1.18 ± 0.04	1.18 ± 0.05	0.834
Ethyl 3-hydroxybutyrate	0.106 ± 0.018	0.090 ± 0.013	0.390	0.15 ± 0.01	0.15 ± 0.02	0.976
Ethyl decanoate	1.46 ± 0.30	1.85 ± 0.22	0.134	0.25 ± 0.01	0.28 ± 0.04	0.594
Isoamyl octanoate	0.005 ± 0.000	0.004 ± 0.001	0.638	1.20 ± 0.32	1.17 ± 0.36	0.812
Ethyl lactate	4.79 ± 1.30	11.2 ± 3.7	0.384	14.1 ± 5.6	10.6 ± 4.5	0.160
Diethyl succinate	0.334 ± 0.089	0.242 ± 0.060	0.282	0.11 ± 0.01	0.20 ± 0.04	0.525
Monoethyl succinate	25.1 ± 5.9	26.3 ± 4.8	0.799	15.3 ± 1.5	19.8 ± 3.1	0.116
**Fatty acids (mg/L)**						
Isobutyric acid	1.36 ± 0.34	1.68 ± 0.13	0.478	1.01 ± 0.30	1.18 ± 0.16	0.601
Butyric acid	2.53 ± 0.55	2.88 ± 0.40	0.297	3.50 ± 0.15	3.42 ± 0.07	0.644
Isovaleric acid	0.814 ± 0.180	0.848 ± 0.096	0.826	0.86 ± 0.08	0.99 ± 0.17	0.379
Valeric acid	0.582 ± 0.040	0.562 ± 0.086	0.863	n.d.	n.d.	--
Hexanoic acid	4.81 ± 0.96	6.98 ± 0.83	0.087	6.84 ± 0.20	7.10 ± 0.29	0.621
Octanoic acid	7.26 ± 1.39	10.4 ± 1.5	0.047	10.8 ± 0.3	10.9 ± 0.7	0.893
Decanoic acid	2.87 ± 0.55	3.70 ± 0.37	0.170	3.66 ± 0.11	3.71 ± 0.32	0.876
Lauric acid	0.290 ± 0.068	0.268 ± 0.037	0.635	0.22 ± 0.03	0.29 ± 0.07	0.524
**Volatile phenols (mg/L)**						
Phenyl acetaldehyde	0.006 ± 0.001	0.008 ± 0.001	0.330	0.011 ± 0.004	0.008 ± 0.002	0.286
β-damascone	n.d.	n.d.	--	0.001 ± 0.000	0.001 ± 0.000	--
Guaiacol	0.020 ± 0.006	0.021 ± 0.009	0.910	0.075 ± 0.033	0.062 ± 0.027	0.642
4-ethyl-guaiacol	0.550 ± 0.220	1.13 ± 0.93	0.410	16.9 ± 1.2	14.6 ± 4.0	0.561
4-vinyl-guaiacol	0.987 ± 0.239	0.808 ± 0.178	0.330	0.930 ± 0.104	0.880 ± 0.089	0.558
Isoeugenol	0.007 ± 0.002	0.005 ± 0.001	0.423	n.d.	n.d.	--
4-vinyl-phenol	3.27 ± 1.15	2.22 ± 0.69	0.432	1.57 ± 0.13	1.61 ± 0.35	0.903
Vanillin	0.023 ± 0.000	0.017 ± 0.002	--	0.008 ± 0.000	0.016 ± 0.000	--
Ethyl vanillate	0.071 ± 0.021	0.066 ± 0.019	0.803	75.6 ± 21.8	75.9 ± 16.2	0.985
**Carbonyl compounds (mg/L)**						
Acetoine	1.40 ± 0.29	6.17 ± 2.33	0.231	2.92 ± 0.82	2.35 ± 0.92	0.675
Furfural	0.118 ± 0.013	1.33 ± 0.90	0.041	0.082 ± 0.013	0.123 ± 0.022	0.502
Benzaldehyde	0.017 ± 0.005	0.015 ± 0.005	0.595	0.008 ± 0.002	0.017 ± 0.003	0.125
**Sulfur compounds (% normalized area)**						
Methionol	30.50 ± 4.39	33.86 ± 5.20	0.278	36.32 ± 5.50	49.28 ± 21.83	0.706
Thiazole	59.39 ± 12.83	63.74 ± 10.07	0.484	10.25 ± 3.22	26.06 ± 6.03	0.331

n.d.: not detected. --: *p*-Value cannot be computed due to lack of data.

## Data Availability

The data presented in this study are available on request from the corresponding author.

## Sample Availability

Samples of the compounds are available from the authors.
